# High-normal albuminuria is associated with subclinical atherosclerosis in male population with estimated glomerular filtration rate ≥60 mL/min/1.73 m^2^: A cross-sectional study

**DOI:** 10.1371/journal.pone.0218290

**Published:** 2019-08-01

**Authors:** Tomoe Kimura, Toshinori Ueno, Shigehiro Doi, Ayumu Nakashima, Toshiki Doi, Aki Ashitani, Reo Kawano, Kiminori Yamane, Takao Masaki

**Affiliations:** 1 Department of Nephrology, Hiroshima University Hospital, Hiroshima, Japan; 2 Center for Integrated Medical Research, Hiroshima University Hospital, Hiroshima, Japan; 3 Nippon Telegraph and Telephone West Corporation, Chugoku Health Administration Center, Hiroshima, Japan; International University of Health and Welfare, School of Medicine, JAPAN

## Abstract

**Background:**

Low-grade albuminuria has been considered a predictor of cardiovascular mortality. We investigated the relationship between high-normal albuminuria and subclinical atherosclerosis in non-diabetic men with estimated glomerular filtration rate (eGFR) ≥60 mL/min/1.73 m^2^.

**Methods:**

In this cross-sectional study, 1,756 men with eGFR ≥60 mL/min/1.73 m^2^ and urine albumin-to-creatinine ratio (UACR) <30 mg/g, who attended general health checkups between April 2012 and March 2015, underwent blood sampling, urinalysis, and carotid ultrasonography. We excluded the subjects who were diabetic and/or received an anti-hypertensive drug. Carotid intima-media thickness (IMT) and the number of focal atheromatous plaques were used as indicators of subclinical atherosclerosis. Multiple linear regression analysis was performed to identify clinical factors associated with carotid IMT. Poisson regression analysis was used to assess the determinants of the carotid plaque number.

**Results:**

Median UACR was 4.8 mg/g (interquartile range, 3.6–6.9 mg/g). Compared with subjects with low-normal UACR (<10.0 mg/g), subjects with high-normal UACR (10.0–29.8 mg/g) had greater IMT and higher carotid plaque number. High-normal UACR was independently associated with thickened IMT in the model adjusted for conventional cardiovascular disease risk factors. Moreover, participants with high-normal UACR were also more likely to be associated with increased plaque count (prevalence ratio: 1.06; 95% confidence interval: 1.01–1.14) after adjustment for conventional cardiovascular disease risk factors.

**Conclusions:**

Our results indicate that high-normal albuminuria is associated with both carotid IMT and plaque formation in the non-diabetic male population with eGFR ≥60 mL/min/1.73 m^2^.

## Introduction

Atherosclerosis is a state whereby the inside of an artery hardens and narrows [1]. Even without symptoms in the early phase, atherosclerosis eventually causes severe cardiovascular disease (CVD) such as coronary heart disease and stroke. Currently the impact of atherosclerosis is estimated at 422.7 million CVD events and 17.9 million CVD deaths worldwide [2], confirming its consideration as a major health problem. According to previous studies, carotid atherosclerosis correlates with not only coronary and cerebral atherosclerosis, but also incidence of CVD [3, 4]. Subsequently, carotid intima-media thickness (IMT) and the extent of atheromatous carotid plaque measured by ultrasonography have become well-established methods for the evaluation of atherosclerosis [5]. In the clinical setting, carotid duplex is mainly performed in subjects with a risk factor for CVD, including smoking, obesity, hypertension, diabetes, and dyslipidemia [6]. Nonetheless many people still die of CVD; therefore, unconventional risk factors should also be determined for the early evaluation of atherosclerosis.

Chronic kidney disease (CKD) has been well recognized as a risk factor for CVD alongside conventional risk factors. In addition to decline in estimated glomerular filtration rate (eGFR) to below 60 mL/min/1.73 m^2^, urinary albumin excretion has also been reported as a strong and independent predictor of CVD mortality [7, 8]. Clinically, the urinary albumin-to-creatinine ratio (UACR) of a spot urine sample has been used to evaluate the daily urinary albumin excretion, and UACR <30 mg/g has been defined as normal. Although normal albuminuria in a population with eGFR ≥60 mL/min/1.73 m^2^ is classified in a low-risk group in the Kidney Disease Improving Global Outcomes heat map [9], recent reports have revealed that, even within normal levels of albuminuria, high-normal albuminuria has emerged as a risk factor for the onset of CVD [8–10]. Additionally, a recent meta-analysis has reported that the cut-off value of UACR that predicts CVD mortality is more than 10 mg/g [8, 9]. In terms of association between UACR and atherosclerosis, previous studies have demonstrated that high-normal albuminuria correlates with IMT [11]. However, the effect of high-normal albuminuria on subclinical atherosclerosis in individuals with eGFR ≥60 mL/min/1.73 m^2^ remains unknown. These findings led us to the idea that determining the association between high-normal albuminuria and carotid atherosclerosis in a healthy population can help prevent a CVD event.

We conducted a cross-sectional study to investigate the associations between the normal range of UACR and subclinical atherosclerosis in Japanese non-diabetic men with eGFR ≥60 mL/min/1.73 m^2^ by performing ultrasonographic assessment of carotid IMT and plaque number. We also compared the thickness of IMT and proportion of plaque number between the populations with low- and high-normal UACR. Moreover, we employed a multivariate regression model to examine whether high-normal albuminuria predicts IMT and plaque number.

## Materials and methods

### Study participants

The subjects included 2,453 Japanese adult males who visited the Nippon Telegraph and Telephone West Corporation Chugoku Health Administration Center (Hiroshima, Japan) for general health checkups between April 2012 and March 2015. We excluded 697 subjects because they met the following exclusion criteria: (1) GFR <60 mL/min/1.73 m^2^ or UACR ≥30 mg/g; (2) hemoglobin A1c (HbA1c) ≥6.5%, 2-h plasma glucose ≥200 mg/dL in the 75-g oral glucose tolerance test, fasting plasma glucose ≥126 mg/dL, or medical history of diabetes [12]; (3) use of antihypertensive drugs affecting excretion of albumin. After exclusion, 1,756 subjects were included in the study. This study was conducted in accordance the Declaration of Helsinki, and the protocol was approved by the ethical committees of the Hiroshima University Hospital (approval number E-1285, registered July 27, 2018). The need for consent was waived by the ethical committees because this was a retrospective cross-sectional study analyzed anonymously and subject management was not adversely affected by the study.

### Anthropometry, blood pressure, and smoking status

Medical information was collected using a standardized questionnaire, which included medical history and lifestyle factors. Height and weight were measured, and body mass index (BMI) was calculated as the weight divided by the height squared (kg/m^2^). Blood pressure (BP) was measured using a mercury sphygmomanometer after 5 min of rest in the sitting position. Hypertension was defined as systolic BP ≥140 mmHg and/or diastolic BP ≥90 mmHg [13]. Current smoking was defined as smoking more than one cigarette per day.

### Laboratory analysis

Urine albumin was measured by means of the latex flocculation immunoturbidimetry assay (Eiken Chemical, Tokyo, Japan). Urine creatinine was examined using an enzymatic method (Eiken Chemical). UACR was calculated by dividing the urine albumin concentration by the urine creatinine concentration, expressed in milligrams per gram (mg/g). Hematuria was defined as more than five red blood cells per high-power field or more than 1+ with the dipstick test for urine. Blood samples were collected following an overnight fast. Serum total cholesterol, triglycerides, high-density lipoprotein (HDL) cholesterol, creatinine, and uric acid levels were analyzed using enzymatic methods (Eiken Chemical). Fasting glucose and HbA1c levels (National Glycohemoglobin Standardization Program equivalent value) were measured by means of high-performance liquid chromatography. Fasting insulin was examined using an enzyme immunoassay (Dainabot, Tokyo, Japan). Insulin resistance was evaluated by homeostasis model assessment of insulin resistance (HOMA-IR), calculated by the formula: (fasting insulin × fasting glucose)/405 [14].

C-reactive protein (CRP) levels were measured using a high-sensitivity, latex-enhanced immunonephelometric assay. eGFR was calculated using the Modification of Diet in Renal Disease equation as follows: eGFR = 194 × Cr^−1.094^ × age^−0.287^ [15]. Low-density lipoprotein (LDL) cholesterol levels were estimated by the Friedewald equation [16]. Dyslipidemia was defined as LDL cholesterol ≥140 mg/dL, HDL cholesterol <40 mg/dL, triglycerides ≥150 mg/dL, or use of lipid-lowering agents [17, 18]. Hyperuricemia was defined as urinary acid ≥7.0 mg/dL or use of antihyperuricemic medications [19].

### Carotid intima-media thickness and plaque number

Carotid ultrasonography was performed by well-trained sonographers following the established method of Pignoli et al. [20], and bilateral carotid arteries were scanned using high-resolution B-mode ultrasonography (EUB-525; Hitachi, Tokyo, Japan, or equivalent apparatus) with the 7.5-MHz probe. We examined the far wall of the left and right common carotid arteries. Examinations were made from three different longitudinal projections (anterior-oblique, lateral, and posterior-oblique). IMT was assessed as the mean value of maximum thickness at any location without involving plaques in the far walls of common carotid arteries on both sides. Most images were analyzed off-line by specifically designed software (Intima-Scope; MEDIA CROSS, Tokyo, Japan). Lesions with a focal IMT of approximately 1.1 mm or greater which has an inflection point on intima-media complex surface were defined as atheromatous plaques. The total number of plaques in the common carotid artery, bulb, and internal and external carotid arteries was counted.

### Statistical analysis

We stratified the subjects into two groups based on UACR level <10.0 mg/g (low-normal UACR) or 10.0–29.9 mg/g (high-normal UACR). Variables are presented as mean ± standard deviation or median and interquartile range (25th–75th percentiles). Differences between groups were compared using the Wilcoxon rank-sum test, Student t-test, or chi-squared test. Multiple regression analyses were used to assess determinants of the thickened IMT, which are presented as β and 95% confidence intervals (95% CI). Poisson regression approaches were used to assess determinants of the increased carotid plaque count, which are presented as prevalence ratio (PR) and 95% confidence intervals (95% CI). Variables included in the analysis were age, BMI, log_10_ CRP, HbA1c, HOMA-IR, eGFR, presence of high-normal UACR, current smoking, hypertension, and dyslipidemia. Statistically significant variables determined in the multivariate regression analysis by stepwise forward selection and backward elimination were subsequently included in a new model. Final analyses also included conventional CVD risk factors. A value of *p* < 0.05 was considered statistically significant. Statistical analyses were performed using JMP 12.0 (SAS Institute, Cary, NC, USA).

## Results

Characteristics of 1,756 men with mean age of 51.5 ± 7.6 years are shown in Table 1. The median UACR of the subjects was 5.1 mg/g (3.6–8.1 mg/g). In the group with high-normal UACR, age (*p* < 0.001), BMI (*p* < 0.001), current smoking rate (*p* = 0.047), systolic BP (*p* < 0.001), diastolic BP (*p* < 0.001), presence of hematuria (*p* < 0.001), as well as the levels of HbA1c (*p* = 0.006), HOMA-IR (*p* < 0.001), urinary acid (*p* = 0.03), total cholesterol (*p* = 0.01), triglycerides (*p* = 0.005), CRP (*p* < 0.001), and eGFR (*p* = 0.012) were all significantly greater than in the group with low-normal UACR.

**Table 1:** Comparison of characteristics between two groups classified by UACR.

Variables	All subjects	Low-normal UACR <10.0 mg/g	High-normal UACR 10.0–29.8 mg/g	*p* Value
Number	1756	1450	306	
UACR, mg/g	5.1 (3.6 to 8.1)	4.4 (3.4 to 6.1)	14.3 (11.6 to 19.8)	<0.001
Age, years	51.5 ± 7.6	51.2 ± 7.7	53.0 ± 7.1	<0.001
BMI, kg/m^2^	23.2 ± 2.8	23.1 ± 2.7	23.9 ± 3.2	<0.001
Current smoking, n (%)	487 (27.7)	388 (26.8)	99 (32.4)	0.047
Systolic BP, mmHg	125.4 ± 13.6	124.1 ± 13.1	131.4 ± 14.5	<0.001
Diastolic BP, mmHg	79.2 ± 9.1	78.4 ± 8.8	83.1 ± 9.6	<0.001
Hematuria, n (%)	147 (8.4)	101 (7.0)	46 (15.0)	<0.001
HbA1c, %	5.7 (5.4 to 5.9)	5.6 (5.5 to 5.9)	5.7 (5.4 to 5.9)	0.006
HOMA-IR	1.2 (0.8 to 1.7)	1.1 (0.8 to 1.6)	1.3 (0.9 to 2.0)	<0.001
Urinary acid, mg/dL	6.1 ± 1.2	6.1 ± 1.2	6.3 ± 1.2	0.03
Total cholesterol, mg/dL	207.0 ± 31.2	206.1 ± 31.0	211.6 ± 31.9	0.01
LDL cholesterol, mg/dL	121.7 ± 28.7	121.3 ± 28.5	123.4 ± 30.0	0.33
Triglycerides, mg/dL	109.0 (76.0 to 157.0)	107.0 (75.0 to 153.0)	119.0 (83.5 to 166.3)	0.005
HDL cholesterol, mg/dL	61.6 ± 16.3	61.4 ± 16.2	62.1 ± 16.9	0.69
CRP, mg/dL	0.04 (0.02 to 0.08)	0.04 (0.02 to 0.07)	0.05 (0.03 to 0.09)	<0.001
eGFR, mL/min/1.73m^2^	73.9 (67.6 to 81.7)	73.7 (67.5 to 81.3)	74.8 (68.9 to 83.3)	0.012

UACR, urine albumin to urine creatinine ratio; BMI, body mass index; BP, blood pressure; HbA1c, hemoglobin A1c; HOMA-IR, homeostasis model assessment of insulin resistance; LDL, low-density lipoprotein; HDL, high-density lipoprotein; CRP, C-reactive protein; eGFR, estimated glomerular filtration rate. Data are expressed as mean ± standard deviation or median (interquartile range) for continuous variables. Differences between groups were compared using Wilcoxon rank-sum test, Student’s t-test, and chi-squared test.

**Fig 1:**
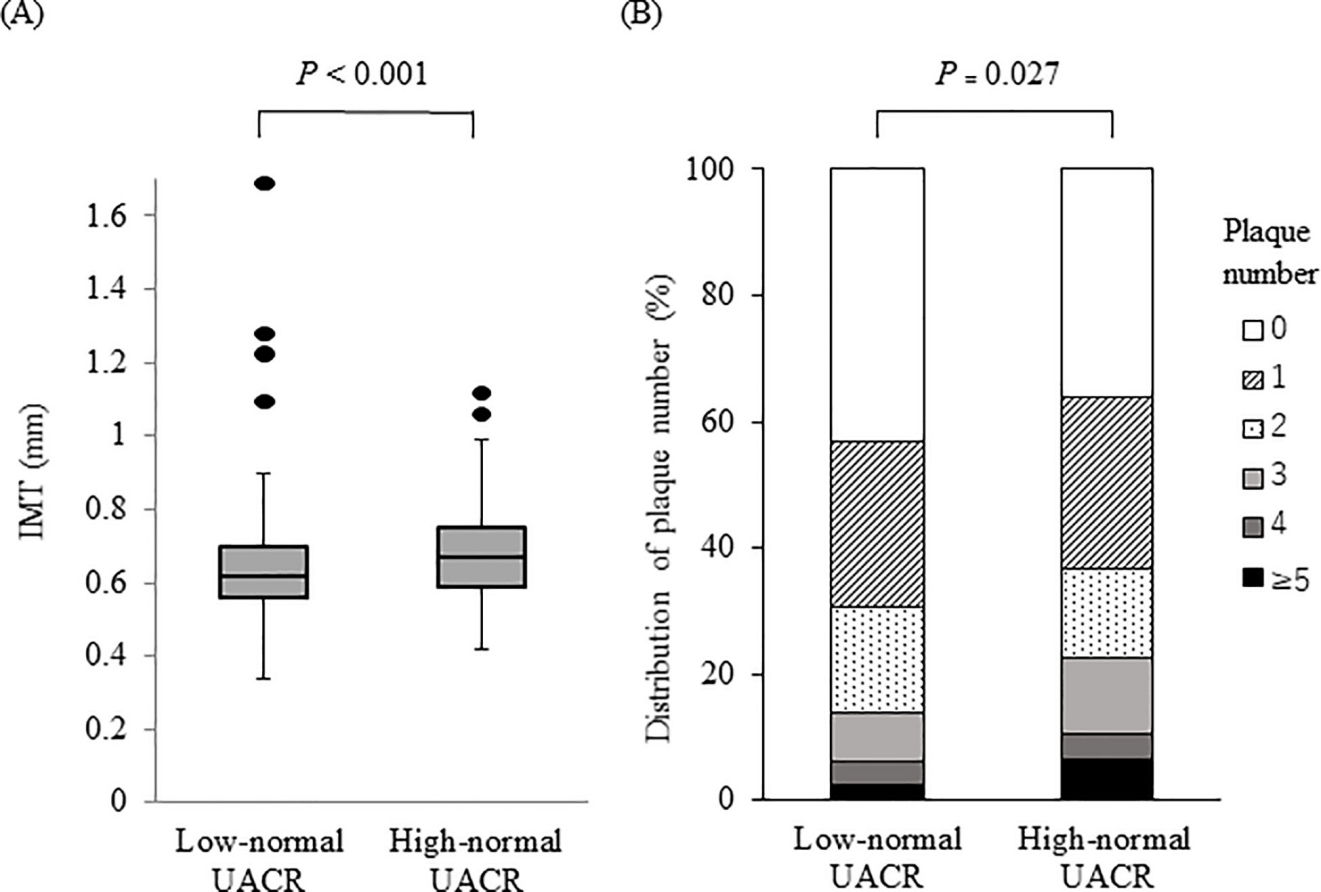
IMT and distribution of carotid plaque number in non-diabetic subjects with normal kidney function. UACR, urine albumin to urine creatinine ratio; IMT, intima-media thickness.(A) The subjects with high normal UACR had significantly greater IMT than those with low-normal UACR (*p* < 0.001). (B) A significant positive correlation with UACR was observed for prevalence of carotid plaque (*p* = 0.027).

The subjects with high-normal UACR had significantly greater IMT (0.62 (0.56–0.70) vs. 0.67 (0.59–0.75), *p* < 0.001) compared with those with low-normal UACR (Fig 1A). Fig 1B shows the comparison between the two groups of distribution of carotid plaque number. Compared with the subjects with low-normal UACR, the prevalence of carotid plaques was significantly higher in those with high-normal UACR (low-normal UACR group, 56.9%; high-normal UACR group, 63.7%; *p* < 0.027). The percentage of subjects who had five carotid plaques or more was also greater in the group with high-normal UACR than in those with low-normal UACR (low-normal UACR group, 2.5%; high-normal UACR group, 6.5%; *p* < 0.001).

Multiple linear regression analysis including conventional CVD risk factors revealed that high-normal UACR (*p* = 0.033) as well as age (*p* < 0.001), BMI (*p* < 0.001), and presence of hypertension (*p* < 0.001) were independently associated with thickened IMT (Table 2).

**Table 2:** Multivariate regression model predicting carotid IMT.

Parameter	β	95% CI	*p* Value
Intercept	0.14	0.072 to 0.209	<0.001
High-normal UACR	0.05	0.0006 to 0.015	0.033
Age, years	0.37	0.006 to 0.007	<0.001
BMI, kg/m^2^	0.18	0.006 to 0.01	<0.001
Current smoking	0.007	−0.005 to 0.007	0.73
Hypertension, presence	0.11	0.011 to 0.025	<0.001
Dyslipidemia, presence	0.01	−0.004 to 0.007	0.50
Log_10_ CRP, log_10_ (mg/dL)	-0.02	−0.02 to 0.006	0.31

The adjusted *r*^2^ of the model was 0.20. UACR, urine albumin to urine creatinine ratio; BMI, body mass index; CRP, C-reactive protein. Hypertension was defined as systolic BP ≥140 mmHg, diastolic BP ≥90 mmHg. Dyslipidemia was defined as LDL cholesterol ≥140 mg/dL, HDL cholesterol <40 mg/dL, triglycerides ≥150 mg/dL, or use of a lipid-lowering drug.

Moreover, Poisson regression approaches revealed that high-normal UACR (PR, 1.06; 95% CI, 1.01–1.14) as well as age (PR, 1.07; 95% CI, 1.06–1.08), presence of hypertension (PR, 1.07; 95% CI, 1.01–1.13), presence of dyslipidemia (PR, 1.07; 95% CI, 1.03–1.12), and log_10_CRP (PR, 1.12; 95% CI, 1.01–1.24) were independent determinants of the increased carotid plaque number (Table 3).

**Table 3:** Multivariate regression model predicting plaque number.

Parameter	PR	95% CI	*p* Value
Intercept	0.05	0.03 to 0.09	<0.001
High-normal UACR	1.06	1.01 to 1.14	0.034
Age, years	1.07	1.06 to 1.08	<0.001
BMI, kg/m^2^	0.99	0.97 to 1.01	0.228
Current smoking	1.05	0.99 to 1.09	0.063
Hypertension, presence	1.07	1.01 to 1.13	0.014
Dyslipidemia, presence	1.07	1.03 to 1.12	0.002
Log_10_ CRP, log_10_ (mg/dL)	1.12	1.01 to 1.24	0.037

PR, prevalence ratio; CI, confidence interval; UACR, urine albumin to urine creatinine ratio; BMI, body mass index; CRP, C-reactive protein. Hypertension was defined as systolic BP ≥140 mmHg, diastolic BP ≥90 mmHg. Dyslipidemia was defined as LDL cholesterol ≥140 mg/dL, HDL cholesterol <40 mg/dL, triglycerides ≥150 mg/dL, or use of a lipid-lowering drug.

Finally, to minimize the bias of exclusion criteria, we have calculated the data without exclusion, assimilating a total of 2,453 study participants. As shown in S1 Table, the subjects with UACR ≥30 mg/g or diabetes had significantly greater IMT and higher carotid plaque number compared with subjects with normal UACR (0–29.9 mg/g) or non-diabetic subjects.

## Discussion

In the present study, we conducted a cross-sectional analysis using data of medical examinations from 1,756 Japanese men with eGFR ≥60 mL/min/1.73 m^2^ and UACR <30 mg/g. We have demonstrated that in this population, high-normal albuminuria is associated with thickened IMT and increased carotid plaque number. Moreover, we show that this association is independent of conventional CVD risk factors. Our results indicate that the population with a UACR of 10–29.9 mg/g exhibit subclinical atherosclerotic changes even though a UACR of less than 30 mg/g is regarded as being low risk for CVD.

As described in the eGFR heat map, decline in renal function is currently recognized as a predictor of not only progression of CKD but also CVD mortality [21, 22]. A decreased number of functional glomeruli induces compensatory increase in the workload of residual glomeruli, contributing to albuminuria through glomerular hyperfiltration. Notably, previous studies have revealed that increasing GFR is also observed in the population with normal kidney function, which is associated with an increase in UACR and incidence of CVD events [23]. In the present study we have demonstrated that subjects with high-normal UACR exhibit higher eGFR than those with low-normal UACR. Given that increased GFR is considered as a state of glomerular hyperfiltration, high-normal albuminuria may reflect glomerular hyperdynamic changes in a population with eGFR ≥60 mL/min/1.73 m^2^.

Glomerular hyperfiltration has been reported in patients with not only diabetes [24, 25] and hypertension [26] but also pre-diabetic or pre-CKD conditions, such as obesity [27]. We have shown here that BMI, BP, HbA1c, HOMA-IR, total cholesterol, and triglycerides in the population with high-normal UACR are significantly higher than those with low-normal UACR. According to past studies, metabolic syndrome increases eGFR through the regulation of system activities of renin-angiotensin-aldosterone and sympathetic nerve, resulting in increased glomerular hyperfiltration [28, 29]. A previous study has reported that metabolic disorder is also known as a risk factor for atherosclerosis [30], and we show here that the subjects with high-normal albuminuria share the same metabolic background as atherosclerosis. These findings suggest that metabolic disorder may participate in the progression of high-normal albuminuria as well as atherosclerosis.

In addition to hyperfiltration, we assume that high-normal albuminuria may be linked to atherosclerosis in terms of endothelial dysfunction. In the glomerular microcirculation, endothelial cells serve as a filtration barrier, and therefore endothelial cell dysfunction, such as loss of the endothelial glycocalyx, contribute to increased albumin excretion [31, 32]. Furthermore, previous studies have revealed that urinary albumin excretion is associated with endothelial dysfunction of the macrocirculation by using flow-mediated dilation of brachial [33] and coronary [34] arteries. Currently, endothelial dysfunction is considered to predispose to greater penetration of atherogenic lipoprotein particles into the arterial wall, playing an important role in the progression of atherosclerosis [32, 35]. Taken together, the association between albuminuria and atherosclerosis might be explained by a common pathophysiologic process of endothelial dysfunction.

Although a number of factors contribute to the pathogenesis of atherosclerosis, we show that, together with age and the presence of hypertension, high-normal UACR independently correlates with both thickened IMT and increased plaque number. Therefore, high-normal UACR is considered to share the same process as IMT thickening and plaque formation. On the other hand, increased BMI correlates with thickened IMT, whereas presence of dyslipidemia and elevation of CRP correlate with plaque number. These results confirm the previous population-based studies showing that obesity is associated with carotid IMT thickening [36], that plasma total cholesterol is strongly associated with lipid core presence in carotid plaque [37], and that CRP is associated with extent of carotid plaque but not IMT [38, 39]. These findings suggest that different pathologic processes may also be implicated in IMT thickening and plaque formation.

This study has several limitations. First, as it was cross-sectional, we were unable to provide an insight into the mechanisms responsible for the observed associations. Second, this study is based on data from records of medical checkups at a company. As a result, the proportion of female participants was small, and we were unable to examine the impact of UACR on subclinical atherosclerosis in women. Third, because the study group consisted of people of Japanese ethnicity, the results need to be replicated in other ethnic groups and should not be generalized without caution. Fourth, we only used a single urine specimen to assess the UACR, which has day-to-day variability [40]. Fifth, as carotid ultrasound was not performed by one sonographer, the influence of the echo technique cannot be completely excluded. Finally, the size of plaque, which is an important factor in plaque formation, was not assessed in this study.

In summary, we found that high-normal albuminuria was associated with subclinical atherosclerosis in a cohort of non-diabetic Japanese men with eGFR ≥60 mL/min/1.73 m^2^ even after adjusting for conventional CVD factors. These results underscore the necessity of UACR screening in medical checkups for the prevention of CVD. Individuals with above high-normal albuminuria require early and active therapeutic intervention for CVD risk factors.

## Supporting information

S1 Table Comparison of carotid IMT and carotid plaque number between two groups classified by UACR and prevalence of diabetes. UACR, urine albumin to urine creatinine ratio; IMT, intima-media thickness. Data are expressed as median (interquartile range) for continuous variables. Differences between groups were compared using Wilcoxon rank-sum test and chi-squared test. ^a^*P*<0.001 vs. UACR 0–29.9 mg/g; ^b^*P*<0.05 vs. UACR 0–29.9 mg/g; ^c^*P*<0.001 vs. non-diabetics; ^d^*P*<0.01 vs. non-diabetics.
(DOCX)
