# Supplementary material for: High-normal albuminuria is associated with subclinical atherosclerosis in male population with estimated glomerular filtration rate ≥60 mL/min/1.73 m2: A cross-sectional study
Source: PLoS One. 2019 Aug 1;14(8):e0218290. doi: 10.1371/journal.pone.0218290 (PMC6675088; doi:10.1371/journal.pone.0218290)
Supplement: S1 Table — UACR, urine albumin to urine creatinine ratio; IMT, intima-media thickness. Data are expressed as median (interquartile range) for continuous variables. Differences between groups were compared using Wilcoxon rank-sum test and chi-squared test. aP<0.001 vs. UACR 0–29.9 mg/g; bP<0.05 vs. UACR 0–29.9 mg/g; cP<0.001 vs. non-diabetics; dP<0.01 vs. non-diabetics. (DOCX) [file pone.0218290.s001.docx]

| **Parameter** | **UACR 0–29.9 mg/g** | **UACR ≥30 mg/g** |  | **Non-diabetes** | **Diabetes** |
| --- | --- | --- | --- | --- | --- |
| Number | 2240 | 213 |  | 2137 | 316 |
| Carotid IMT, mm | 0.63 (0.57 to 0.72) | 0.69 (0.61 to 0.78)^a^ |  | 0.63 (0.57 to 0.72) | 0.68 (0.59 to 0.77)^c^ |
| Prevalence of carotid plaques, n (%) | 1362 (60.8) | 155 (72.3)^a^ |  | 1290 (60.3) | 227 (71.8)^c^ |
| Carotid plaque number, n (%) |  |  |  |  |  |
| 1 | 591 (26.4) | 47 (22.1) |  | 569 (26.6) | 69 (21.8) |
| 2 | 384 (17.1) | 48 (22.5)^b^ |  | 365 (17.1) | 67 (21.2) |
| 3 | 214 (9.6) | 28 (13.2) |  | 197 (9.2) | 45 (14.2)^d^ |
| 4 | 98 (4.4) | 13 (6.1) |  | 86 (4.0) | 25 (7.9)^d^ |
| ≥5 | 75 (3.3) | 19 (8.9)^a^ |  | 73 (3.4) | 21 (6.6)^d^ |

**S1 Table.** **Comparison of carotid IMT and carotid plaque number between two groups classified by UACR and prevalence of diabetes**

UACR, urine albumin to urine creatinine ratio; IMT, intima-media thickness. Data are expressed as median (interquartile range) for continuous variables. Differences between groups were compared using Wilcoxon rank-sum test and chi-squared test. ^a^*P*<0.001 vs. UACR 0–29.9 mg/g; ^b^*P*<0.05 vs. UACR 0–29.9 mg/g; ^c^*P*<0.001 vs. non-diabetics; ^d^*P*<0.01 vs. non-diabetics.

.
